# In-Hospital Mortality Risk Factor Analysis in Multivessel Percutaneous Coronary Intervention Inpatient Recipients in the United States

**DOI:** 10.7759/cureus.17520

**Published:** 2021-08-28

**Authors:** Ravi Tummala, Suchi D Shah, Era Rawal, Ramneek K Sandhu, Swathi P Kavuri, Gagan Kaur, Asma T Khan, Keerthika Mathialagan, Temitope Ajibawo

**Affiliations:** 1 Internal Medicine, Narayana Medical College, Nellore, IND; 2 Internal Medicine, Ahmedabad Municipal Corporation's Medical Education Trust Medical College, Ahmedabad, IND; 3 Cardiology, Norvic International Hospital, Kathmandu, NPL; 4 Internal Medicine, Sri Guru Ram Das Institute of Medical Sciences and Research, Amritsar, IND; 5 Internal Medicine, Sri Ramachandra Institute of Higher Education and Research, Chennai, IND; 6 Surgery, Sri Guru Ram Das Institute of Medical Sciences and Research, Amritsar, IND; 7 Internal Medicine, Larkin Community Hospital, South Miami, USA; 8 Psychiatry, Sree Balaji Medical College and Hospital, Chennai, IND; 9 Internal Medicine, Brookdale University Hospital Medical Center, New York City, USA

**Keywords:** acute myocardial infarction, primary pci, primary percutaneous coronary intervention (pci), in-hospital mortality, nationwide inpatient sample (nis), perioperative mortality

## Abstract

Objectives

The primary goal of our study is to evaluate the mortality rate in inpatient recipients of multivessel percutaneous coronary intervention (MVPCI) and to evaluate the demographic risk factors and medical complications that increase the risk of in-hospital mortality.

Methods

We conducted a cross-sectional study using the Nationwide Inpatient Sample (NIS, 2016) and included 127,145 inpatients who received MVPCI as a primary procedure in United States' hospitals. We used a multivariable logistic regression model adjusted for demographic confounders to measure the odds ratio (OR) of association of medical complications and in-hospital mortality risk in MVPCI recipients.

Results

The in-hospital mortality rate was 2% in MVPCI recipients and was seen majorly in older-age adults (>64 years, 74%) and males (61%). Even though the prevalence of mortality among females was comparatively low, yet in the regression model, they were at a higher risk for in-hospital mortality than males (OR 1.2; 95% CI 1.13-1.37). While comparing ethnicities, in-hospital mortality was prevalent in whites (79%) followed by blacks (9%) and Hispanics (7.5%). Patients who developed cardiogenic shock were at higher odds of in-hospital mortality (OR 9.2; 95% CI 8.27-10.24) followed by respiratory failure (OR 5.9; 95% CI 5.39-6.64) and ventricular fibrillation (OR 3.5; 95% CI 3.18-3.92).

Conclusion

Accelerated use of MVPCI made it important to study in-hospital mortality risk factors allowing us to devise strategies to improve the utilization and improve the quality of life of these at-risk patients. Despite its effectiveness and comparatively lower mortality profile, aggressive usage of MVPCI is restricted due to the periprocedural complications and morbidity profile of the patients.

## Introduction

Annually, over 790,000 acute myocardial infarctions (AMI) occur in the United States (US), and the number one cause of death remains coronary artery disease [1]. Percutaneous coronary intervention (PCI) became a standard of treatment for revascularization in patients with ischemic heart disease since the 1970s [1]. Every year, over 1,000,000 PCIs are performed worldwide [2]. It is the preferred technique under numerous circumstances, including high-surgical risk patients and multivessel disease with a low syntax score [3]. PCI is also less economically taxing than its more invasive counterparts. It is, therefore, no surprise that the rate of growth of PCI centers in the US has bypassed its population growth rate [4].

Multivessel percutaneous coronary intervention (MVPCI) procedures are done predominantly in the elderly population. Males undergo MVPCI procedures twice compared to females and this predominance is likely because of a lower prevalence of coronary diseases among women of similar age [5]. Racial differences play an integral part in MVPCI utilization. Among the ethnic minorities, blacks are the least likely to utilize resources like PCI or coronary artery bypass graft (CABG), where differences in clinical presentation and physicians' bias in treating minorities contribute to the discrepancy [6].

Patients undergoing MVPCI have baseline comorbidities, most notably hypertension and diabetes. However, the percentage of MVPCI utilization in diabetic patients decreased by 40.5% from 2006 to 2012 [7]. The drastic decrease in the utilization of MVPCI is due to a higher mortality rate compared with CABG. However, in other instances, MVPCI is similar in efficacy to PCI, and reclassification of treatment in 2015 by the cardiology board has led to further use [7].

However, like any medical intervention, PCI does pose certain risks of morbidity and mortality. Early complications of MVPCI are stent thrombosis, bleeding, coronary dissection, and renal failure. Late complications are complications of coronary heart disease. The Cleveland Clinic institutional PCI registry report found that 2% of mortality post-PCI occurred within the first 30 days. Of these deaths, 58% died of cardiac and 42% of non-cardiac causes. Interestingly, less than half (42%) of 30-day deaths were attributed to PCI-related complications [8]. Another study found that the major causes of death within the first 30 days after undergoing PCI were cardiogenic shock and anoxic brain injury post-cardiac arrest [9]. However, MVPCI in patients with cardiogenic shock following AMI had a significantly better one-year outcome (21.3%) than patients undergoing single-vessel PCI (31.7%) [10]. Age, culprit vessel size and flow, and the presence of heart failure and diabetes were determined to be risk factors and independent predictors of mortality [11].

So, we conducted a cross-sectional inpatient study to evaluate the mortality rate in patients hospitalized and managed with MVPCI and to evaluate the demographic predictors and medical conditions that increase the risk of in-hospital mortality in MVPCI recipients.

## Materials and methods

Study sample

We conducted a cross-sectional study using the Nationwide Inpatient Sample (NIS) from January 1 to December 31, 2016. We were not required to take the institutional review board's permission for this study as the NIS is a de-identified publicly available dataset and protects any health-related patient information [12]. We included 127,145 adult patients who were hospitalized and were undergoing MVPCI treatment as the primary procedure.

Variables

Demographic variables included were age at admission (<44, 45-64, or >64 years), sex (male or female), and race/ethnicity (white, black, Hispanic, or others). Comorbid medical complications included in the study are cardiogenic shock, ventricular fibrillation (VFib), respiratory failure, intracranial hemorrhage (ICH), periprocedural stroke, acute kidney injury (AKI), and acute gastrointestinal (GI) bleeding. Our primary hospital outcome is in-hospital mortality, which is all-cause mortality in the NIS [13].

Statistical analysis

We compared the distributions of demographic characteristics, and comorbid medical complications by in-hospital mortality using descriptive statistics and Pearson’s chi-square test. Next, we used a logistic regression model adjusted for demographic confounders to measure the odds ratio (OR) of association of comorbid medical complications and in-hospital mortality risk in inpatients undergoing MVPCI. All analyses were conducted using SPSS Statistics v. 26.0 (IBM Corp., Armonk, NY) and statistical significance was set at a two-sided P-value <0.05.

## Results

We included 127,145 medical inpatients who underwent MVPCI as a primary procedure during hospitalization; the in-hospital mortality rate was 1.88%. A higher proportion of these inpatients who died during hospitalization were older-age adults above 64 years of age (74.4%) and males (60.8%). While comparing ethnicities, in-hospital mortality was most prevalent in whites (79.0%), subsequently blacks (8.8%), Hispanics (7.5%), and other races (4.6%). The most prevalent comorbid medical complications among patients who died during hospitalization were a respiratory failure (60%), followed by AKI (53.2%), cardiogenic shock (49.9%), and VFib (37.3%) as shown in Table 1.

**Table 1:** Distribution of inpatients undergoing multivessel percutaneous intervention by in-hospital mortality

Variable	In-hospital mortality		P-value
	No (in %)	Yes (in %)	
Number of inpatients	124,760	2,385	-
Age at admission			
<44 years	5.2	2.3	<0.001
45-64 years	41.2	23.3	
>64 years	53.7	74.4	
Sex			
Male	64.4	60.8	<0.001
Female	35.6	39.2	
Race			
White	75.4	79.0	<0.001
Black	10.4	8.8	
Hispanic	7.5	7.5	
Others	6.8	4.6	
Comorbid medical complications			
Cardiogenic shock	2.6	49.9	<0.001
Ventricular fibrillation	7.2	37.3	<0.001
Respiratory failure	6.3	60.0	<0.001
Acute kidney injury	13.6	53.2	<0.001
Acute gastrointestinal bleeding	0.5	5.7	<0.001
Periprocedural stroke	0	0.2	0.02
Intracranial hemorrhage	0	0	-

Even though the prevalence of in-hospital mortality among females was comparatively low, females were at a higher risk of mortality than males (OR 1.24; 95% CI 1.13-1.37). Compared to the white population, other races/ethnicities had a lower and non-significant association with in-hospital mortality.

There existed a statistically significant association between certain risk factors and in-hospital mortality. Patients who developed cardiogenic shock were at higher odds of mortality (OR 9.20; 95% CI 8.27-10.24), followed by respiratory failure (OR 5.99; 95% CI 5.39-6.64), acute gastrointestinal (GI) bleeding (OR 3.67; 95% CI 2.83-4.75), VFib (OR 3.53; 95% CI 3.18-3.92), and AKI (OR 2.49; 95% CI 2.26-2.75) as shown in Table 2.

**Table 2:** In-hospital mortality risk predictors in inpatients undergoing multivessel percutaneous intervention

Variable	Logistic regression model		P-value
	Odds ratio	95% confidence interval	
Age at admission			
<44 years	Reference		
45-64 years	0.99	0.73-1.36	0.988
>64 years	2.44	1.80-3.31	<0.001
Sex			
Male	Reference		
Female	1.24	1.13-1.37	<0.001
Race			
White	Reference		
Black	0.94	0.79-1.11	0.471
Hispanic	1.12	0.93-1.33	0.229
Others	0.62	0.49-0.77	<0.001
Comorbid medical complications			
Cardiogenic shock	9.20	8.27-10.24	<0.001
Ventricular fibrillation	3.53	3.18-3.92	<0.001
Respiratory failure	5.99	5.39-6.64	<0.001
Acute kidney injury	2.49	2.26-2.75	<0.001
Acute gastrointestinal bleeding	3.67	2.83-4.75	<0.001
Periprocedural stroke	8.95	1.15-69.52	0.036
Intracranial hemorrhage	-	-	-

## Discussion

In our comprehensive analysis study of 127,145 inpatient recipients of MVPCI, we determined demographic predictors (age, gender, and race) and medical risk factors that increase the risk of in-hospital mortality. Several papers have already shown the mortality in MVPCI is comparable or even lower than that of post-PCI mortality. Our study further analyzes the potential benefits of MVPCI by assessing in-hospital mortality in the demographic and comorbidity subcategories.

Whites had the highest in-hospital mortality rate in our study inpatients. An observational study in 2019 evaluated race and gender disparities in MVPCI outcomes and resource utilization. Two important conclusions that they made were that the black population had the highest prevalence of comorbidities and Asian women had the highest risk of in-hospital mortality, with rates of in-hospital morbidities increasing yearly with the largest increment in the Hispanic population [6]. Contrary to past findings, we found post-MVPCI hospitalizations more prevalent in whites, which could be due to higher utilization of cardiovascular procedures in general among this population [14].

In-hospital mortality was significantly higher in females by 24% compared to males. Females tend to have a higher mean low-density lipoprotein (LDL) than men because of physiologically higher estrogen levels, wider use of hormonal contraceptives, and hormone replacement therapy contributing to a higher comorbid profile [15]. Other factors like smaller body size, limited access to the quality of care, and inadequate usage of cardioprotective medicines could also contribute to the discrepancy [16]. Gender disparities in terms of preference for cardiovascular testing have also led to seeking healthcare at an advanced stage of the disease, which can directly alter the mortality with reperfusion [17].

Advanced age is a universal predictor of death in cardiovascular diseases and procedures [18]. The extensive coronary atherosclerosis, physiological stiffening leading to decreased left ventricular systolic and diastolic function, and tortuous vascular anatomy pose an added risk. A higher baseline comorbidity profile and impaired age-related compensation make it a treatment-risk paradox [19]. We do not have proper data of their pre-existing comorbid profile, but studies show significant improvement with emergency revascularization (utilizing MVPCI) for mitigation of complications, especially in global diffuse coronary artery disease [20].

A recent meta-analysis confirms that multivessel disease is common in ST-elevated myocardial infarction (STEMI) patients [21]. Theoretically, MVPCI can reperfuse hibernating and stunned myocardium, thereby improving ventricular function. However, our study shows an increased association with mortality. It could be due to increased procedural time, contrast use, and inflammatory milieu due to hemodynamic insult [22]. Studying the outcomes of MVPCI can help devise strategies to treat versus not to treat non-culprit vessels. Untreated non-culprit vessels can lead to long-term rehospitalization, especially for subsequent revascularization for heart failure [23].

Our study shows that VFib was an important risk factor for mortality, owing to the prothrombotic and proinflammatory nature of an acute disease like STEMI [24]. Global reperfusion would help in alleviating any future infarction due to the instability of the lesion. But it is a double-edged sword as it depends on many factors like onset to balloon time, levels of b-type natriuretic peptide, sympathetic tone, elevated lactate/phosphate levels, and many more factors [25]. This again suggests that it is an interplay, and hence reperfusion cannot be a standalone factor for mortality [26].

Periprocedural stroke is clinically significant and a disabling risk factor for mortality due to micro-embolisms leading to anoxic brain injury. Increased contrast load and multiple ischemic insults due to shock led to renal failure as well. Our study confirms that GI bleed was one of the significant and probably preventable causes of mortality following MVPCI. Pre-existing peptic ulcer disease (PUD), increased vagal stimulation following shock causing ulcers that bleed off, and concomitant usage of antiplatelet therapy could be risk factors besides physiological stress to GI mucosa [27].

There are certain limitations associated with the study conducted. Firstly, this is a cross-sectional study and thus it is hard to establish causal relationships for in-hospital mortality risk, and it is reported all-cause in the NIS. Furthermore, the database does not contain in-depth patient-level clinical information, and in this study, the relevant data were extracted using diagnostic codes, which may have caused the under-reporting of comorbidities. Yet, there are some strengths of this study. NIS database is a large inpatient population data that lead to uniform patient records and provide a population-based perception of mortality associated with systematic and temporal factors. Further, the information is coded independently by the individual physicians, which protects it from reporting bias.

## Conclusions

Accelerated use of MVPCI made it important to study in-hospital mortality risk factors allowing us to devise strategies to improve the utilization and improve the quality of life of these at-risk patients. The highest in-hospital mortality following MVPCI was among females and whites. MVPCI inpatients with medical complications had a higher risk of mortality, with cardiogenic shock posing the highest risk ( increased by nine times), followed by respiratory failure (increased by six times), and VFib (increased by 3.5 times). Despite its effectiveness and comparatively lower mortality profile, aggressive usage of MVPCI is restricted due to the periprocedural complications and morbidity profile of the patients.
